# Cardiac Arrest and Complete Heart Block: Complications after Electrical Cardioversion for Unstable Supraventricular Tachycardia in the Emergency Department

**DOI:** 10.3390/jpm14030293

**Published:** 2024-03-09

**Authors:** Adina Maria Marza, Claudiu Barsac, Dumitru Sutoi, Alexandru Cristian Cindrea, Alexandra Herlo, Cosmin Iosif Trebuian, Alina Petrica

**Affiliations:** 1 Department of Surgery, “Victor Babes” University of Medicine and Pharmacy, 300041 Timisoara, Romania; marza.adina@umft.ro (A.M.M.); dumitru.sutoi@umft.ro (D.S.); alexandru.cindrea.umfvbt@gmail.com (A.C.C.); trebuian.cosmin@umft.ro (C.I.T.); alina.petrica@umft.ro (A.P.); 2 Emergency Department, Emergency Clinical Municipal Hospital, 300079 Timisoara, Romania; 3 Clinic of Anaesthesia and Intensive Care, “Pius Brinzeu” Emergency Clinical County Hospital, 300736 Timisoara, Romania; 4 Department of Infectious Diseases, “Victor Babes” University of Medicine and Pharmacy, 300041 Timisoara, Romania; alexandra.mocanu@umft.ro; 5 Department of Anesthesia and Intensive Care, Emergency County Hospital, 320210 Resita, Romania; 6 Emergency Department, “Pius Brinzeu” Emergency Clinical County Hospital, 300736 Timisoara, Romania

**Keywords:** supraventricular tachycardia, cardioversion, unstable patient, complete heart block, ineffective pacing, cardiac arrest, advanced life support

## Abstract

Synchronous electrical cardioversion is a relatively common procedure in the emergency department (ED), often performed for unstable supraventricular tachycardia (SVT) or unstable ventricular tachycardia (VT). However, it is also used for stable cases resistant to drug therapy, which carries a risk of deterioration. In addition to the inherent risks linked with procedural sedation, there is a possibility of malignant arrhythmias or bradycardia, which could potentially result in cardiac arrest following this procedure. Here, we present a case of complete heart block unresponsive to transcutaneous pacing and positive inotropic and chronotropic drugs for 90 min, resulting in multiple cardiac arrests. The repositioning of the transcutaneous cardio-stimulation electrodes, one of them placed in the left latero-sternal position and the other at the level of the apex, led to immediate stabilization of the patient. The extubation of the patient was performed the following day, with full recovery and discharge within 7 days after the insertion of a permanent pacemaker.

## 1. Introduction

Patients with symptomatic supraventricular arrhythmias commonly present in the emergency department (ED), necessitating urgent management. Sporadically, complications may arise, but there is a lack of comprehensive literature regarding post-cardioversion events for supraventricular tachycardia (SVT).

Traditionally, SVT encompasses all tachycardias except for ventricular tachycardias (VTs) and atrial fibrillation (AF), with atrial rates exceeding 100 beats per minute at rest [1]. Narrow QRS tachycardia is defined as a QRS duration of 120 ms or less, while sometimes, they may display a widened QRS complex exceeding 120 ms due to pre-existing conduction delays either related to heart rate or to bundle branch blocks [1,2,3].

In the general population, the prevalence of SVT is 2.25 per 1000 individuals, with an incidence of 35 per 100,000 person-years [1]; evidence from the United States indicates that they contribute to approximately 50,000 ED visits each year [4]. However, epidemiological studies on SVT populations are limited. Women face twice the risk of developing SVT compared to men, while individuals aged 65 years or older have over five times the risk compared to their younger counterparts [1]. Additionally, SVT is also frequent in patients with congenital heart disease [5].

The clinical presentations of SVT vary extensively, spanning from mild symptoms like palpitations and breathing disturbances to severe symptoms associated with hemodynamic instability and cardiogenic shock, which pose a risk to the patient’s life [6,7]. The assessment and management of all arrhythmias consider both the patient’s condition and the characteristics of the arrhythmia. The goal is to prevent cardiac arrest.

Therefore, the initial approach in the ABCDE assessment involves identifying the patient’s adverse features, such as shock, syncope, acute heart failure, and myocardial ischemia. Additionally, it is essential to consistently monitor the heart rhythm and blood pressure and administer oxygen if SpO2 falls below 94%. Identifying and addressing reversible causes, such as electrolyte imbalances or hypovolemia, should be conducted in accordance with the 2021 European Resuscitation Council (ERC) Guidelines [8]. If these signs are absent in a patient with regular tachycardia, vagal maneuvers will be performed [9], with the inverted Valsalva maneuver demonstrated to be more efficient in adults [1]. In the ED, the standard protocol includes conducting a thorough history, physical examination, and a 12-lead ECG, supplemented by usual laboratory tests such as full blood counts, biochemistry profile, and thyroid function assessment. If feasible, transthoracic echocardiography should also be conducted.

If vagal maneuvers fail to resolve the issue, the initial drug of choice is adenosine (6–12–18 mg) [1,8]. A recently published study by Xiao et al. compared the efficiency of Valsalva maneuvers, adenosine, and their combination, but the results were inconclusive [10]. If adenosine proves ineffective, intravenous verapamil, diltiazem, or beta-blockers are the subsequent treatment options for narrow complex SVT, while intravenous procainamide or amiodarone are recommended for wide complex SVT. Given that atrioventricular node blockers are contraindicated for patients with pre-excited atrial fibrillation [11], the differential diagnosis should be meticulously performed before their administration. If these treatments are ineffective, synchronized cardioversion of up to three attempts is advised to terminate the tachycardia. Having a stable patient provides the advantage of granting the emergency physician the opportunity to seek expert advice for both the differential diagnosis of SVT and its treatment if initial measures prove unsuccessful.

For unstable patients exhibiting life-threatening features, synchronized cardioversion under sedation is the treatment of choice [12]. If unsuccessful, administering intravenous amiodarone at a dose of 300 mg over 10–20 min or intravenous procainamide at a dose of 10–15 mg/kg over 20 min is recommended, followed by repeating synchronized shocks, if necessary [1,8].

Here, we describe a case of a patient requiring electrical cardioversion for SVT. Following the synchronized shock, the patient experienced severe bradycardia, subsequently progressing to complete heart block unresponsive to medication and pacing, culminating in cardiorespiratory arrest.

## 2. Case Presentation

A 53-year-old male patient, a known smoker with no other comorbidities except for a right bundle branch block (RBBB), arrived at the ED via a physician-staffed ambulance. The patient presented with main complaints of palpitations and fatigue that started approximately 8 h before arrival, along with diaphoresis and dyspnea exacerbated by minimal efforts, particularly in the last hour.

**Figure 1:**
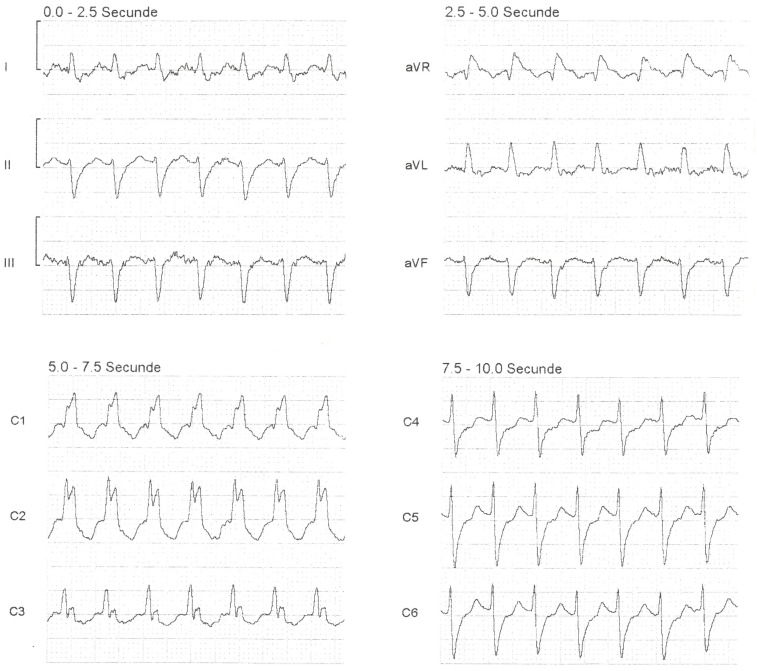
A 12 lead-ECG from the ambulance: regular rhythm tachycardia (heart rate 174 BPM) and wide QRS complexes of 0.14 s, with rSR’ pattern in V1 and V2; findings indicative of supraventricular tachycardia (SVT) with right bundle branch block (RBBB).

Before reaching the hospital, a 12-lead ECG (Figure 1) and vital signs monitoring were conducted, revealing SVT with RBBB, a heart rate of 174 BPM, SpO2 at 98%, and blood pressure of 130/80 mmHg. Deeming the patient’s condition stable, the emergency physician attempted vagal maneuvers, which had no effect on the heart rate. Adenosine was administered in three doses (6 mg, 12 mg, 18 mg), also with no impact on the patient’s condition. After a 30 min period during which 10 mg of metoprolol was slowly intravenously administered without success, the decision was made to transfer the patient to the ED.

**Figure 2:**
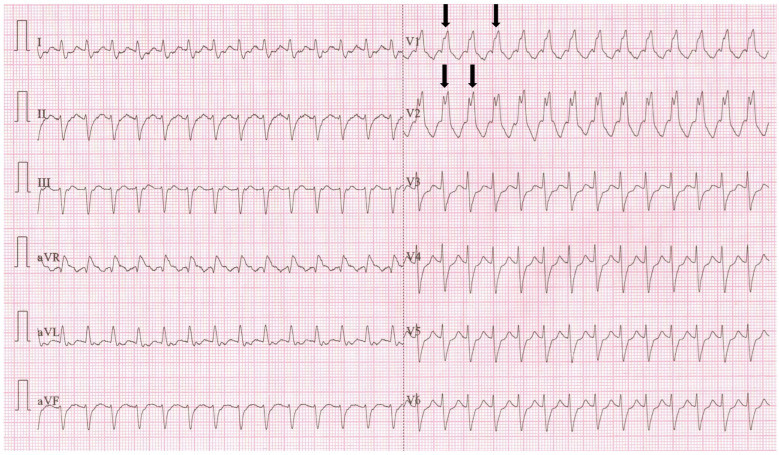
First 12-lead ECG (recorded at 25 mm/s, and a voltage of 10 mm/mV) from ED—regular rhythm tachycardia (heart rate 174 BPM), QRS duration of 0.14 s, rSR’ pattern in V1 and V2 (arrows), absent P waves, negative T waves in V1 and V2; findings suggestive for SVT with RBBB.

Upon arrival at the ED, the patient appeared conscious and cooperative, but he was pale, diaphoretic, and exhibited dyspnea at rest. Vital signs indicated a peripheral oxygen saturation of 93%, blood pressure at 90/60 mmHg, a heart rate of 174 BPM, a capillary refill time of 3 s, and a barely detectable radial pulse. The ECG showed SVT with RBBB, as seen in Figure 2.

The patient was administered supplemental oxygen at a rate of 3 L/min; blood work-up and arterial blood gas analysis were conducted (Table 1). After approximately 30 min, it was decided that synchronous cardioversion with 70 joules was the optimal course of action. The patient provided consent for the procedure and was sedated beforehand.

**Figure 3:**
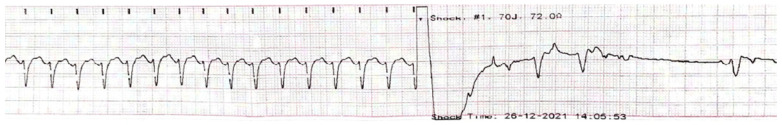
The defibrillator’s rhythm recording during the electrical cardioversion shows irregular bradycardia after the synchronous shock for SVT.

Following the initial synchronous shock, the patient experienced severe bradycardia (Figure 3) for a few seconds, transitioning into asystole. Immediate resuscitation measures, including chest compressions and bag-mask ventilation, were initiated. After about 30 s, the patient exhibited spontaneous breathing and movement of limbs, opened his eyes, and responded to verbal stimuli.

**Figure 4:**
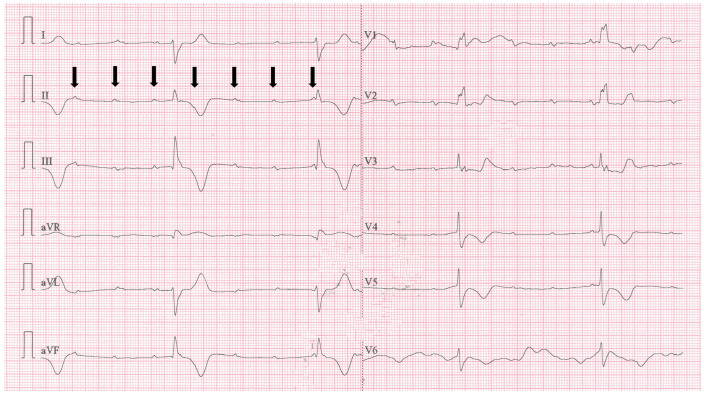
A 12-lead ECG (recorded at 25 mm/s, and a voltage of 10 mm/mV) after ROSC shows atrio-ventricular dissociation, with a ventricular heart rate of 27 BPM and QRS duration of 0.17 s, regular P waves (arrows), variable PR interval, right axis deviation, negative T waves in DII, DIII, aVF, V4–V6 and ST depression in the V3–V5 leads. The findings are consistent with a complete heart block and myocardial ischemia in the infero-lateral territory.

A repeated ECG recording revealed a third-degree AV block (Figure 4), with a heart rate of 23 BPM, and the patient was hemodynamically unstable. Intravenous Atropine 0.5 mg was administered, followed by additional doses every 2 min, up to a maximum of 3 mg. Simultaneously, Adrenaline was administered via a syringe infusion pump with 2 mcg/min, with no improvement in the patient’s condition. Transcutaneous pacing was initiated with a frequency set to “Demand” at 70 BPM, 80 mA intensity. Although the monitor indicated efficient capture, the femoral pulse was not concordant. Pacing parameters were adjusted to 70 BPM with an increase in intensity up to 160 mA and even 200 mA for short periods of time, yet the myocardium remained unresponsive to external pacing.

**Figure 5:**
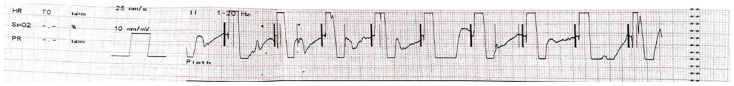
Transcutaneous pacing with ineffective capture and inconsistent femoral pulse. Pacing settings: mode—demand, frequency—70 BPM, intensity—160 mA.

Despite efforts to improve hemodynamic stability, the transcutaneous pacing remained ineffective (Figure 5). The patient’s thorax was shaved to enhance transcutaneous conduction, and the initial electrode position (right subclavian/cardiac apex) was changed to an antero-posterior and, later, to a latero-lateral position. The Adrenaline dose was increased from 2 mcg/min to 20 mcg/min. Additionally, alternative medications, including Aminophylline 24 mg over 10 min and Dopamine via a syringe infusion pump at 10 mcg/kg/min, were administered.

Throughout the patient’s stay in the ED, he experienced cardiopulmonary arrest at least 10 times, manifesting as either asystole or pulseless electrical activity. Each time, the patient responded positively to external thoracic compressions, mechanical ventilation, and Adrenaline administration, achieving a return of spontaneous circulation in less than 2 min.

**Figure 6:**
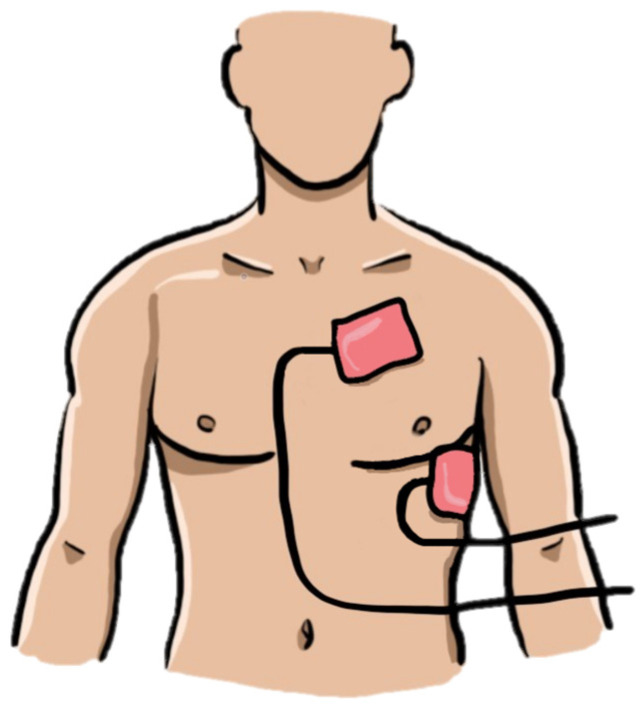
Placement of both transcutaneous stimulation electrodes on the left hemithorax: subclavicular left (latero-sternal) and cardiac apex (replacing ECG lead V6) positions.

The on-call physician at the regional Institute for Cardiovascular Diseases was contacted and agreed to admit the patient once his condition was stable for transport. Despite exhausting all available treatment options in the ED (which lacked equipment for transvenous pacing), the patient continued to experience severe hypotension between cardiopulmonary arrest episodes due to the unresponsiveness to transthoracic cardio-stimulation. Considering the inefficacy of pacing, attributable to the patient’s thoracic anatomy with a large anterior-to-posterior diameter, a decision was made to reposition the transthoracic pacing leads to subclavicular left (latero-sternal) and cardiac apex (replacing ECG lead V6) positions (Figure 6). Upon reassessment, the change in lead position resulted in successful pacing capture, confirmed by femoral artery pulse and an increase in blood pressure values.

Approximately 90 min after the initial cardioversion attempt, the patient’s stability allowed for transportation to the regional Institute for Cardiovascular Diseases for transvenous pacing. The final evaluation indicated a blood pressure of 115/50 mmHg, heart rate of 70 BPM, consistent femoral pulse, GCS of 3 with the patient mechanically ventilated under continuous sedative medication and receiving inotropic and vasopressor medication (Adrenaline and Dopamine). Emergency transthoracic echocardiography showed a left ventricle of normal size with preserved systolic function, an ejection fraction of 50%, medio-basal hypokinesia of the lateral wall, concentric left ventricular hypertrophy, grade II mitral and tricuspid regurgitation, medium secondary pulmonary hypertension, dilated right ventricle, and a posterior pericardial fluid blade measuring below 1 cm.

Upon arrival at the regional Institute for Cardiovascular Diseases, the patient’s condition improved with the placement of the transvenous pacing probe, later replaced with a permanent pacemaker implant. The following day, the patient was extubated, demonstrating complete hemodynamic and neurologic recovery, and was discharged from the hospital a week later.

## 3. Discussion

Synchronized cardioversion, a potentially life-saving procedure, is commonly utilized in the emergency department for hemodynamically unstable tachyarrhythmias. Emergency physicians should possess a thorough understanding of the indications and potential complications before recommending or performing this intervention. The effectiveness of frequently utilized anti-arrhythmic drugs is restricted in these situations, primarily due to concerns over side effects and their potential to induce arrhythmias, thereby limiting their usage [13].

When selecting the appropriate method for cardioversion, it is important to follow the current guidelines and carefully weigh the potential benefits and risks associated with each intervention. Sometimes, the patient is at the threshold of receiving either electrical or drug cardioversion and requires a prompt decision to distinguish between the two, given that both interventions carry inherent risks. Ventricular fibrillation (VF) as a complication of electrical cardioversion was documented in numerous studies, particularly when cardioversion takes place during the vulnerable period of repolarization, typically around the peak of the T wave on the ECG [14,15]. In 2004, Lyndon and Abdul described such a case, highlighting the significance of adjusting the lead configuration on the defibrillator monitor to prevent mistaking the high T wave for the R wave, thereby averting the induction of VF during the administration of synchronous external electric shock [15]. Kaufmann et al. described two cases of iatrogenic ventricular fibrillation after the cardioversion of pre-excited atrial fibrillation due to inadvertent T-wave synchronization [16].

In this scenario, the patient was presented with recent-onset palpitations lasting several hours, and appropriate drug therapy had already been administered in prehospital settings. Given the unavailability of intravenous verapamil or diltiazem in the region, the only remaining option for terminating the tachycardia was electrical cardioversion. Considering the patient’s known history of right bundle branch block, the tachycardia was managed as SVT. Chronologically, the decision to proceed with electrical cardioversion was made over an hour after the last bolus of metoprolol, during which time no significant reduction in heart rate was observed.

Severe bradycardia, immediately followed by complete heart block and subsequent cardiac arrest with pulseless electrical activity, was an unexpected and rarely reported complication of synchronized cardioversion. Similar outcomes were described by Gallagher et al., who conducted a retrospective cohort study examining the relationship between shock energy and arrhythmic complications of electrical cardioversion. Sinus bradycardia or a slow junctional escape rhythm was observed in 22 cases, with 20 resolving within minutes after cardioversion. While two patients required permanent pacing before hospital discharge, neither needed rate support while awaiting pacemaker implantation. None of these patients experienced cardiac arrest. They also found that the incidence of ventricular fibrillation (VF) following shocks of <200 J was significantly higher compared to higher energy shocks (5 out of 2959 vs. 0 out of 3439 shocks, *p* = 0.021). Additionally, non-sustained broad complex tachycardia occurred in four cases, all lasting less than 10 s: two after shocks > 200 J and two after shocks less than 200 J. The induction of atrial fibrillation (AF) was significantly more common with shocks of <200 J (20 out of 930 shocks vs. 1 out of 313 shocks at ≥200 J, *p* = 0.015) [17].

The success of cardioversion relies on several factors, with time being the most crucial. In this case, the patient experienced symptoms for several hours, and the initial EKG recording, which revealed SVT with a heart rate of 174, was conducted more than three hours prior to arriving at the ED. Nevertheless, the patient initially declined to come to the hospital. The prolonged duration of the tachyarrhythmia could potentially lead to both post-repolarization and conduction delays due to global ischemia, as described in other studies, which reported VF as a complication following cardioversion for AF [18,19].

Furthermore, a proposed theory regarding the mechanism of complete heart block in this patient was the administration of intravenous metoprolol before cardioversion, given that beta blockers are known as drugs with sinoatrial and/or atrioventricular nodal-blocking properties [20]. In a retrospective and prospective study conducted by Osmonov et al. involving 108 patients treated with atrioventricular blockers and presenting symptomatic type II second- or third-degree AV block, 2:1 AV block, atrial fibrillation, and bradyarrhythmia, it was found that 36 patients treated with metoprolol experienced metoprolol-induced AV blocks that persisted or recurred in 24 patients [21].

However, the maximum therapeutic dose of 15 mg (recommended by ESC guidelines, American College of Cardiology/American Heart Association Task Force on Clinical Practice Guidelines, and the Heart Rhythm Society for stable SVT) [1,20] was not achieved in this case; only 10 mg was administered intravenously in 2.5 mg boluses, with no discernible effect on heart rate following the last bolus. This hypothesis was considered due to the lack of effectiveness of the transcutaneous pacing, assuming the patient had a stronger response to beta-blockers. Unfortunately, glucagon, the antidote for beta-blocker overdose, was unavailable at the time in any of the hospitals in the area, preventing the assessment of this hypothesis.

Also, myocardial ischemia or metabolic disturbances such as acidosis and hypoxia were described as factors that can elevate the pacing threshold and potentially prevent capture [22]. The patient experienced both metabolic acidosis and severe hypoxemia in the period following cardioversion, conditions that were rectified only after successful cardiac pacing.

Successful capture is typically identified by a widened QRS complex, succeeded by a clear ST segment and broad T wave. A pulse rate manually confirmed in the femoral artery or right carotid artery notably lower than the pacing rate displayed on the pacing unit monitor may suggest a lack of capture [22].

In this case, achieving efficient capture required placing the electrodes closer together. While we cannot definitively assert that this positional change was the sole factor stabilizing the patient, given the limited application to only one patient, it proved to be the sole measure in a unique and critical situation that yielded an immediate positive effect, leading to a sudden improvement in the patient’s condition. Consequently, we cannot consistently advocate or recommend this procedure in routine practice. However, in similar situations where other well-known methods prove ineffective and the patient’s condition continuously deteriorates, as exemplified in this case report, it may be considered a life-saving measure.

## 4. Conclusions

Cardiac arrest and complete heart block are uncommon complications following electrical cardioversion. Given the infrequency of capture failure cases with transcutaneous pacing, addressing each isolated case can provide significant benefits to both the ED and prehospital staff, particularly in the management of atypical situations.

## Figures and Tables

**Table 1:** Pathological values of the laboratory tests performed upon arrival in the ED.

Laboratory Test	Value	Reference Range Value	Conventional Units
Blood glucose	146	74–106	mg/dL
Creatinine	1.52	0.70–1.30	mg/dL
D-dimer	1.89	<0.68	mg/L
Lactate	2.52	0.36–0.75	mmol/L
NT-pro-BNP	10,217	<125	pg/mL
Troponin I	58.2	17–50	ng/L
White blood cells	11.1	4.0–10.0	×10^9^/µL

Abbreviations: NT-pro-BNP, N-terminal pro-B-type natriuretic peptide.

## Data Availability

The data presented in this study are available upon request from the corresponding author.
